# A refined magnetic pulse treatment method for magnetic navigation experiments with adequate sham control: a case study on free-flying songbirds

**DOI:** 10.1098/rsif.2023.0745

**Published:** 2024-05-15

**Authors:** Thiemo Karwinkel, Michael Winklhofer, Dario Allenstein, Vera Brust, Paula Christoph, Richard A. Holland, Ommo Hüppop, Jan Steen, Franz Bairlein, Heiko Schmaljohann

**Affiliations:** ^1^ Institute of Avian Research ‘Vogelwarte Helgoland’, An der Vogelwarte 21, 26386 Wilhelmshaven, Germany; ^2^ School of Mathematics and Science, Institute of Biology and Environmental Sciences, Carl von Ossietzky Universität Oldenburg, Ammerländer Heerstraße 114–118, Oldenburg 26129, Germany; ^3^ Research Center for Neurosensory Sciences, Carl von Ossietzky Universität Oldenburg, Ammerländer Heerstraße 114–118, Oldenburg 26129, Germany; ^4^ Institute of Landscape Ecology, Westfälische Wilhelms-Universität Münster, Heisenbergstr. 2, Münster 48149, Germany; ^5^ School of Environmental and Natural Sciences, University of Bangor, Deiniol Road, Bangor LL57 2UW, UK; ^6^ Max Planck Institute of Animal Behavior, Am Obstberg 1, Radolfzell 78315, Germany

**Keywords:** bird navigation, magnetic-particle-based sensor, magnetic map, magnetic pulse, bird migration, trigeminal magnetic sensor

## Abstract

Migratory songbirds may navigate by extracting positional information from the geomagnetic field, potentially with a magnetic-particle-based receptor. Previous studies assessed this hypothesis experimentally by exposing birds to a strong but brief magnetic pulse aimed at remagnetizing the particles and evoking an altered behaviour. Critically, such studies were not ideally designed because they lacked an adequate sham treatment controlling for the induced electric field that is fundamentally associated with a magnetic pulse. Consequently, we designed a sham-controlled magnetic-pulse experiment, with sham and treatment pulse producing a similar induced electric field, while limiting the sham magnetic field to a value that is deemed insufficient to remagnetize particles. We tested this novel approach by pulsing more than 250 wild, migrating European robins (*Erithacus rubecula*) during two autumn seasons. After pulsing them, five traits of free-flight migratory behaviour were observed, but no effect of the pulse could be found. Notably, one of the traits, the migratory motivation of adults, was significantly affected in only one of the two study years. Considering the problem of reproducing experiments with wild animals, we recommend a multi-year approach encompassing large sample size, blinded design and built-in sham control to obtain future insights into the role of magnetic-particle-based magnetoreception in bird navigation.

## 1. Introduction

Migratory birds use, besides other cues (e.g. stars and sun), the Earth's geomagnetic field for orientation and navigation during their seasonal journeys [1]. Whereas ‘orientation’ refers to a compass direction [2], ‘navigation’ implies a position determination system, enabling goal-finding from places that were previously unknown to the birds [3]. How birds sense the required magnetic information is unclear. It is commonly assumed that they possess two independent magnetic sensors [4]. The magnetic compass sensor is most likely based on the influence of the magnetic field on light-dependent molecular reactions in the retina, often called the radical-pair sense [5]. The second type is thought to be located in the beak and based on magnetic particles, i.e. the magnetic-particle sensor [6]. The best evidence for the involvement of magnetic particles in magnetoreception comes from studies with birds that reoriented in circular orientation cages (Emlen funnels) after exposure to a brief but strong magnetic pulse, aimed at switching the magnetization of the particles [7–16]. Only adult birds were affected by such a pulse pre-treatment, whereas pulse-treated juveniles kept their seasonally appropriate compass orientation [12,13]. For this, juveniles rely only on the radical-pair based magnetic compass, which is not affected by magnetic pulsing [5]. The reported pulse effects on adult birds only suggest that the magnetic-particle sensor serves the geomagnetic map, i.e. location information.

The geomagnetic map hypothesis relies on the fact that the Earth's magnetic field varies systematically over the globe, thereby providing a useful source of spatial information [17]. The hypothesis also depends on the assumption that birds imprint on the geographical variation of the Earth's magnetic field during their first outbound migration towards their non-breeding areas and later extrapolate spatial gradients to determine their position [18,19]. Accordingly, experienced birds that artificially experienced the geomagnetic field of an unfamiliar location by virtual displacement [18,20,21] or were physically translocated to outside the individual’s known route [19,22], were found to correct their displacements. This would also explain why the magnetic-particle sensor is affected by the magnetic pulse treatment only in adults, performing true navigation, but not in juveniles, which on their first migration have to first learn the map and orient only by a compass [2].

Despite the physical plausibility of the magnetic-particle hypothesis of magnetoreception, a candidate anatomical structure for such a magnetoreceptor has been found only in rainbow trout (*Oncorhynchus mykiss*) [23,24] but remains elusive in birds [25,26]. Due to the lack of structural information, we can only speculate in what way a magnetic pulse would affect the sensor. The pulse may (1) remagnetize the polarity of putative single-domain particles in the direction of pulsing, as shown in rainbow trout [24], (2) temporarily alter the particle arrangement [15,27–29], or (3) disable the sensor by disrupting the putative biological structure containing the magnetic particles. Whichever the effect, migratory birds should perceive artificially altered or faulty geomagnetic map information that could lead to various behavioural responses, including impaired decision-making for when and in which direction to resume migration (e.g. disorientation).

The vast majority of studies on songbirds that observed a pulse effect were performed under controlled conditions in orientation cages (Emlen funnels) in settings similar to magnetic compass experiments [7–16]. By contrast, only a handful of pulse experiments were conducted with freely moving animals [28–32], and these yielded incongruent results: reorientation [28,29], or no effects at all [30,31] ([32] is inconclusive due to late departures of birds).

The observed effects of magnetic pulsing on orientation behaviour have been interpreted in terms of magnetic-particle-based magnetoreception in the context of detecting magnetic map information, although the pulse also includes a non-magnetic treatment. The most pertinent non-magnetic effect of such a pulse is the induced electric field, which may affect the electric state of neurons. Exactly this effect is harnessed in transcranial magnetic stimulation (TMS), where a magnetic pulse is applied to electrically stimulated cortical areas in a non-invasive way through the induced electric field. In high-intensity TMS, the magnetic field typically changes within 0.2 ms from 0 to 3 T [33] and is sufficient to evoke action potentials, to the point of eliciting involuntary muscle contractions when delivered over the motor cortex [33–35]. While pulsing devices used in magnetic orientation studies produced induced voltages about 30 times smaller compared to a high-intensity TMS device, these are still comparable in magnitude to those applied in modest intensity (i.e. sub-motor threshold) repetitive magnetic stimulation techniques, which in turn have been shown to alter cortical excitability [36]. Thus, electric side effects due to even a moderately strong pulse on the bird brain cannot be ruled out and need to be controlled for in magnetoreception studies if behavioural effects of the pulse are to be attributed to the specific action of the magnetic field on a magnetic-particle magnetoreceptor.

We addressed this limitation of previous pulse studies by designing a pulsing device that can fire a treatment pulse and a sham-control pulse. The latter reaches only a tenth of the magnetic field intensity of the treatment pulse, but otherwise produces the same induced electric field as in the treatment. This design therefore controls for potential effects of the induced electric field. We applied our refined and novel pulsing treatment to wild European robins (*Erithacus rubecula*). We chose this species because it strongly responded to a pulse pre-treatment in the wild on autumn migration, resulting in disorientation at the group level [29]. To account for the variable results of free-flight pulse studies (see above), we chose a large sample size and, for the first time in pulse studies, a 2-year approach. We also used an advanced automated regional-scale radio-tracking system with high spatial and temporal resolution to observe any effects on behavioural levels of birds' free-flight with a blinded analysis [30,31]. If our treatment pulse produces any effects, i.e. compared to the sham control, we can reject the idea that the induced electric field is responsible for pulse effects and favour the alternative hypothesis, that the magnetic field is the effector. This would, for the first time, provide convincing evidence for magnetic pulse effects favouring the magnetic-particle-based hypothesis of magnetoreception.

## 2. Methods

### 2.1. Study site and species

**Figure 1.**
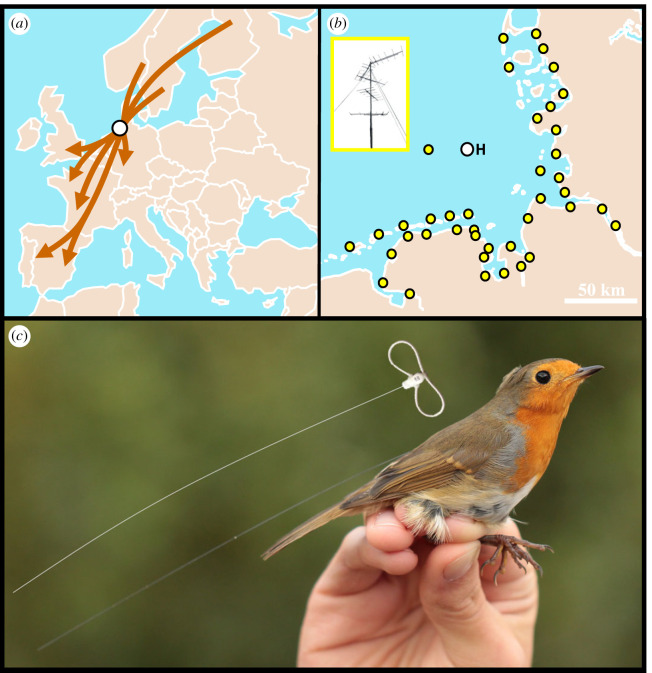
Tracking system for European robins (*Erithacus rubecula*). (*a*) Map showing presumed autumn migration routes of robins passing the island of Helgoland (white dot) according to [37,38]. (*b*) Map showing locations of radio-receiving stations (yellow dots) around the study site of Helgoland (white dot). Inset displays an example radio-receiving antenna site. (*c*) Robin with attached radio transmitter and radio transmitter with leg-loop harness illustratively shown above the bird. Photo: T.K. Geographical data for maps were downloaded from the GSHHG database of the NOAA (https://www.ngdc.noaa.gov/mgg/shorelines/).

The study was conducted on Helgoland (54.18 N, 7.89 E; a small isolated island in the German Bight of the North Sea, figure 1*a,b*) during autumn migration 2020 and 2021. The study species, the European robin (*Erithacus rubecula*, ‘robin’ hereafter), is abundant on the island during migration, coming mostly from the Fennoscandian breeding region and heading to wintering grounds mainly from central to southwest Europe (figure 1*a*) [37,38]. Robins did not breed on Helgoland in 2020 [39] and 2021 [40]. Thus, all robins caught on the island were active migrants, which rested, fuelled and recovered there between their migratory flights [41]. During a stopover, migrants must decide from night to night whether to depart and if so, when and in which direction to take off. Due to Helgoland's distant offshore location, we are convinced that every robin leaving the island resumed migration, cf. [42] and did not perform local landscape movements. Robins migrate at night [43] and, most likely, independently of conspecifics.

For this experiment, we caught 258 robins with Helgoland funnel traps, mist nets or mealworm-baited spring traps between 21 September and 7 November 2020 (118 birds) and between 18 August and 7 November 2021 (140 birds). Birds were aged as first-year birds (juveniles hereafter, *n* = 129) or older birds (adults hereafter, *n* = 129) based on plumage characteristics and the coloration of the inner upper mandible. Maximum wing length was measured [44]. After catching, the birds were housed indoors in individual cages at the island station of the Institute of Avian Research ‘Vogelwarte Helgoland’, and were provided with food (mealworms, *Tenebrio molitor*, supplemented with fat-food, Fett-Alleinfutter Typ II grün, Claus GmbH, Germany) and water ad libitum for a few days.

### 2.2. Experimental procedure

**Figure 2.**
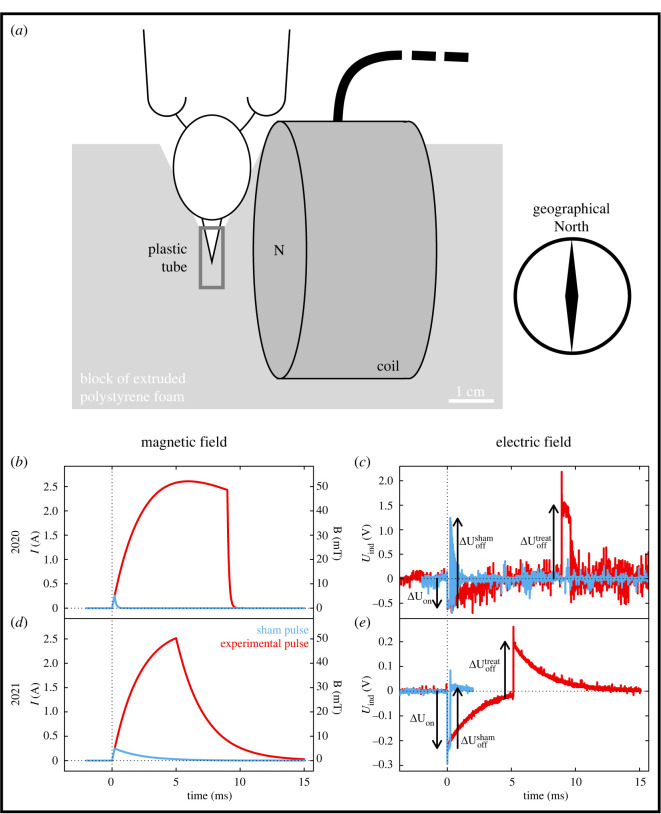
Application and electromagnetic properties of the pulse. (*a*) For pulsing, the bird was handheld in an indentation of an extruded polystyrene foam block with a small plastic tube for fixation of the beak, ensuring consistent pulse application geometry for every bird. The coil produced the electromagnetic pulse with its magnetic north pointing perpendicular to the beak. The beak pointed geographically southwards. The bird's beak was exposed to a peak magnetic field intensity of approximately 52 mT in the treatment group and to approximately 5.2 mT in the sham control group. Magnetic field (*b*,*d*) and induced voltage (*c*,*e*) course of the sham control (blue) and treatment (red) pulse in the study years of 2020 (*b*,*c*) and 2021 (*d*,*e*). The induced voltage in 2021 was measured with a smaller pickup coil (approximately half the effective area compared to 2020) to avoid the ringing (fast oscillations in blue trace of (*c*), which is why the vertical scale for *U* in (*e*) is smaller compared to (*c*)). For a given pick-up coil, Δ*U*_on_ is the same for both pulsing devices.

For the experiment, we only chose days for which migration-favourable weather conditions were expected in the following night, i.e. no rain and wind speeds of about less than 8 m s^−^^1^ [45]. On those days, we assigned equal numbers of housed robins to the treatment and sham control groups. The birds were weighed to the nearest 0.1 g and muscle score [46] was assigned for calculating fuel load [47]. The pulse application took place outdoors on a wooden table about 6 h before sunset, as an endocrinological study suggested that migratory songbirds make their departure decision for the upcoming migratory flight a few hours before sunset [48]. The birds were individually exposed to either the treatment or sham control pulse by holding them next to the coil of the pulser (figure 2*a*). We adjusted the distance between the pulse coil and the beak of the bird such that the peak field was approximately 52 mT for the treatment pulse (figure 2*a*), sufficient to significantly remagnetize any known population of biogenic single domain magnetite [27]. In particular, it would suffice to completely remagnetize the candidate magnetoreceptor structure studied in [24], whose magnetic polarity switched in a field of about 20 kA m^−1^ (25 mT), but was not affected by fields below 10 kA m^−1^ (12 mT) (figure 4 of [24] for the magnetization curves). For the sham control pulse, fired at the same distance as the treatment pulse, the peak field measured 5.2 mT, i.e. lower than any known switching field of biogenic single domain magnetite [27]. The peak magnetic field of the pulse was measured with a Hall probe (Gaussmeter HGM09 s, MAGSYS Magnet Systeme GmbH, Dortmund, Germany).

Immediately after pulse application, birds were fitted with a leg-loop harnessed radio transmitter (NTQB-2, Lotek Wireless Inc., Canada; approximately 0.31 g including harness, figure 1*c*) and released into the wild. An automated radio receiving array on Helgoland [49] and along the coast of the German Bight [30,50] (figure 1*b*), recorded the signals which were transmitted with a time resolution of 2.3 to 5.3 s. Recordings were processed as part of the Motus Wildlife Tracking System [51] to assign the radio signals to the uniquely coded transmitters, i.e. to individual birds. The detection data were then analysed, similar to our former studies [30,31], with an algorithm, determining (I) departure probability, (II) stopover duration, (III) departure timing within the night, (IV) departure direction off Helgoland for the first approximately 10 km and (V) consistency of the flight direction until passage at the shoreline (for more information on these traits, see the electronic supplementary material). A person who did not participate in the fieldwork and had no information regarding to which group the birds belonged validated the data manually and assisted the algorithm wherever needed. In doing so, we ensured a maximum blinding procedure during data analysis.

### 2.3. Concept of the sham control pulse

A magnetic pulse, by definition, is a time-dependent magnetic field B(t), which according to Faraday's law of electromagnetic induction, generates an induced voltage $U_{\mathrm{ind}}(t)$ proportional to the rate of change, −dB/dt. A magnetic pulse thus always elicits a voltage spike at the onset of the pulse (t=0) and typically a second spike of the opposite sign when it is switched off at $t=t_{d}$. To realise a sham control for the voltage spikes generated in the treatment pulse without changing the geometry of the set-up (i.e. same coil, same circuit), we designed a sham control pulse, with the same initial dB/dt as the treatment pulse, but of short enough duration $t_{d,\mathrm{Sh}}$ (e.g. 0.2 ms) to limit the sham magnetic field to a tenth of the peak magnetic field of the treatment pulse (with $t_{d,\mathrm{Tr}}$ of *ca* 5 to 10 ms). By doing so, we produced a sham control pulse with comparable induced voltage spikes as in the treatment pulse, while limiting the peak field of the sham pulse to 10% of the treatment pulse.

### 2.4. Pulsing devices

For the first field season (autumn 2020), we used the commercial magnetic pulser MP 09 (e-Med Instruments, London, UK), which we modified such that the duration of the magnetic pulse (initially preset to 12 ms) was adjustable between $t_{d,\mathrm{Tr}}=9$ ms for the treatment pulse and $t_{d,\mathrm{Sh}}=0.2\;$ms for the sham control pulse. When the pulse is fired at t=0, the current in the pulse coil rises at an initial rate of

2.1 $${\left(\frac{dI}{dt}\right)}_{t=0}=\frac{U_{0}}{L},$$

where *L* is the inductance of the pulse coil (170 mH, measured with an LCR bridge, Fluke Corp., Everett, WA, USA, at 1 kHz) and $U_{0}$ is the initial voltage (230 V) provided by a capacitor (C=270 μF) starting to discharge at t=0. The numerical value of $U_{0}/L$ amounts to 1.3 A ms^−1^ and the current-to-field conversion ratio at the chosen distance corresponds to 24 mT A^−1^.

At $t_{d,\mathrm{Sh}}=0.2$ ms, the current has reached 0.24 A, and is shut off in the sham control pulse. By contrast, the current in the treatment pulse is allowed to reach its peak of 2.4 A (at 5.8 ms) and shut off at $t_{d,\mathrm{Tr}}=9$ ms (figure 2*b*). Since the magnetic field B(s) at any given distance *s* from the coil is proportional to the coil current, the magnetic treatment pulse is 10 times stronger than the sham control pulse at the same distance *s*. Similarly, since the rate of change in the magnetic field is proportional to that of the current (equation (2.1)), both pulses at their onset produce the same induced voltage spike according to Faraday's law (figure 2*c*). A second spike is produced when the coil current is switched off: now the current decreases from 2.25 A to 0 A within 0.69 ms (−3.2 A ms^−1^, treatment), and from 0.24 A to 0 A within 0.1 ms (−2.4 A ms^−1^, sham control), thereby producing turn-off spikes that exceed the turn-on spikes in magnitude.

For the second field season, we kept using the MP 09 coil but designed our own magnetic pulse circuitry aimed at reducing the magnitude of the turn off spike by slowing down the field decay and because the originally modified device got damaged. The core circuit (electronic supplementary material, figure S1) consists of two capacitors (470 µF, model MAL215759471E3, Vishay Inc., Malvern, PA, USA) connected in parallel, as a source of fast electric power, and a high-power switch (*R*_on_ = 0.024 Ω, power MOSFET model IXTK120N25P, IXYS GmbH, Lampertheim, Germany). The capacitors are charged by a DC voltage source (*U*_0_ = 230 V) via a 1000 Ω resistor. To produce a pulse, a +5 V_DC_ control signal is applied to the gate of the power MOSFET for a duration of $t_{D}$, adjustable from 0.1 to 10 ms with a GUI interface on a laptop computer. Once the switch is activated, the capacitors start discharging and the coil current increases at an initial rate of 1.3 A ms^−1^ (see equation (2.1)) to reach a maximum of $I_{\max}=2.65\;A$ at *ca* 8 ms (if $t_{D}\geq$ 8 ms, else at $t_{D}$). At $t=t_{D}$, the switch is deactivated and the magnetic energy stored in the coil is drained through a flyback diode (SiC Schottky Diode, FFSH5065A, ON Semiconductor, Hong Kong, China), whereby the coil current (and magnetic field) decays exponentially with a relaxation time of $\tau =L/R_{L}$ (*ca* 2.2 ms (figure 2*d*), where $R_{L}$ (*ca* 80 Ω) is the equivalent series resistance of the coil. The spike amplitudes produced when the coil current is switched off now amount to 0.11 A ms^−1^ (sham control) and 1.1 A ms^−1^ (treatment) and thus are smaller than the spike at the onset of each pulse (1.3 A ms^−1^; figure 2*e*). When firing the pulse, no matter whether sham control or treatment, the magnetic field (at the location of the bird's head, approximately 52 mT for treatment pulse) initially rose at a rate of 30 mT ms^−1^.

### 2.5. Revisiting pulsing devices used in previous studies

For comparison, we recorded pulses generated by the original devices used in previous pulse studies on free-flying birds (see electronic supplementary material, table S1). The Sota pulsing device, with its custom-made double-wrapped solenoid with 10 cm diameter clearance [28,29,32,52], produced a treatment pulse with peak amplitude slightly over 0.1 T (with 50% peak reached within 0.1 ms), which agrees with the description in [52]. In control mode, where the pulse currents in the double-wrapped solenoid flow in opposite directions through its two windings and cancel out each other's magnetic fields [52], the coil leaked a pulse of 1 mT strength and thus generated a measurable induced electric field, albeit significantly smaller than in the treatment mode (electronic supplementary material, table S1). Therefore, the control mode in the double wrapped coil is not an adequate control for the treatment mode in terms of induced electric fields. The leakiness of the double-wrapped pulse solenoid results from incomplete field cancellation of the windings in antiparallel current mode.

The pulse used in indoor orientation experiments on songbirds was described to produce a *ca* 4 ms long magnetic pulse with 0.5 T peak amplitude [14,15], but neither pulse shape nor coil inductance was reported. Assuming the pulse reached half peak within 0.5 ms, the rate of change would amount to 0.5 T ms^−1^, comparable to the values for the Sota pulser (electronic supplementary material, table S1).

### 2.6. Statistics

All statistical analyses were performed using R v. 4.0.3 software [53]. For testing effects on departure probability, we used Pearson's *χ*^2^-test. Stopover duration and departure timing were compared using Wilcoxon rank sum test [53]. Whenever ties (the same value multiple times) in the data prevented the Wilcoxon rank sum test from calculating exact *p*-values, we switched to the exact Wilcoxon rank sum test, embedded in the R package ‘exactRankTests’ [54]. For circular data, we used tests embedded in the R packages ‘CircStats’ [55] and ‘circular’ [56]. As our circular data points had ties, we repeated the Mardia–Watson–Wheeler test 10 000 times to exclude any effect of random tie breaking and provide the median of the test parameters. The Mardia–Watson–Wheeler test can test for differences in mean angle, angular variance or both simultaneously [57]. We included the dataset and the R code of the statistical analyses in the electronic supplementary material.

For two reasons, we avoided using statistically more complex models (e.g. linear mixed effect models) to assess the effect of the treatment on the birds' behaviour, although they would allow including biologically relevant intrinsic and extrinsic factors: first, our experimental procedure with simultaneous releases standardized all extrinsic parameters between the groups, such as weather conditions. Additionally, feeding the caged birds prior to their release standardized intrinsic factors, like fuel load, which did not differ between experimental and sham control groups (*t*-test: juveniles *t* = −0.12, *p* = 0.90; adults *t* = −0.83, *p* = 0.41). Second, as models must be fitted to individual datasets, using models would have prevented us from performing the exact same test for subsets of data, necessary for table 1. That table demonstrates how different the outcome of the experiment would be when performed only in 1 year or independent of age group. We compared the intrinsic and extrinsic conditions between the 2 years and found no biologically relevant differences (electronic supplementary material, table S2).

**Table 1.** Testing the effect of an electromagnetic pulse on five free-flight behavioural levels of European robins (*Erithacus rubecula*) on different subsets of the data. Numbers give the *p*-value of the corresponding test, testing pulse treated against the sham control group. *p*-Values of less than 0.05 are marked with an asterisk.

	2020			2021			2020 + 2021		
	juveniles	adults	juv.+ad.	juveniles	adults	juv.+ad.	juveniles	adults	juv.+ad.
departure probability	0.344	0.042*	0.021*	0.928	1.000	1.000	0.378	0.205	0.099
stopover duration	0.354	0.050*	0.027*	0.700	0.889	0.713	0.382	0.179	0.089
departure timing	0.599	0.291	0.237	0.218	0.528	0.175	0.207	0.181	0.061
departure direction	0.313	0.175	0.060	0.762	0.893	0.930	0.328	0.505	0.207
consistency in flight	0.504	0.210	0.571	0.047*	0.222	0.095	0.049*	0.126	0.204

## 3. Results

The results presented here pool the data of both study years. Year-specific analyses are provided in table 1, figure 4, electronic supplementary material, figure S3 and the supplemented R code.

**Figure 3.**
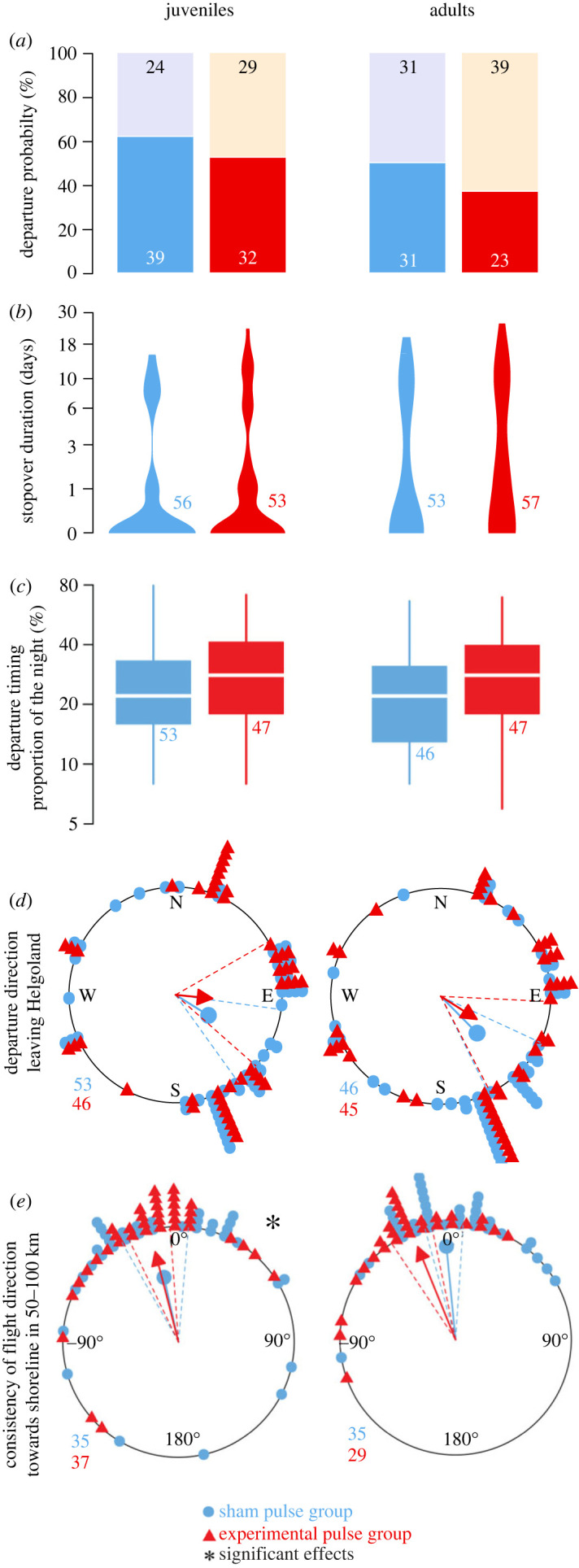
Effects of an electromagnetic pulse on five migratory traits in juvenile and adult free-flying European robins (*Erithacus rubecula*) aggregated from 2020 and 2021 autumn migration. The graphics for the individual years can be found in figure 4 and electronic supplementary material, figure S3. Numbers are sample sizes (individual birds). Sample sizes decrease from *a–e* as not every trait could be assigned for every bird; see methods for details. (*a*) Departure probability represents the proportion of birds staying for the first night after treatment and release (upper bar, black numbers) or departing on the first night from Helgoland (lower bar, white numbers). (*b*) Distribution of stopover duration after treatment and release. (c) Nocturnal departure timing as proportion of the night length. Only birds departing in the first 10 days were considered for this and the following traits. (*d*) Initial departure direction from Helgoland. (*e*) The consistency of the initial direction until passage at the shoreline of the German Bight (50–100 km, figure 1*b*). Data points in the circular plots are shifted slightly by less than 5° to better distinguish both groups.

Departure probabilities of juveniles in the sham control (62%, *n* = 63) and treatment group (52%, *n* = 61) did not differ (Pearson's *χ*^2^-test: $\chi_{1}^{2}=0.777$, *p* = 0.378; figure 3*a*). We also found no effect in adults (sham control: 50%, *n* = 62, treatment: 37%, *n* = 62; Pearson's *χ*^2^-test: $\chi_{1}^{2}=1.607$, *p* = 0.205; figure 3*a*; table 1). When testing the effect independent of age class, no effect could be found (Pearson's *χ*^2^-test: $\chi_{1}^{2}=2.723$, *p* = 0.099, *n* = 248, table 1).

**Figure 4.**
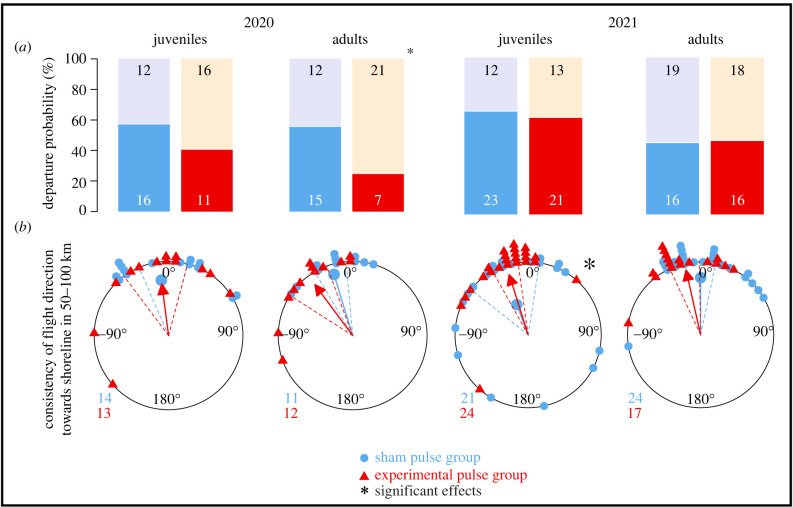
Non-reproducible effects of an electromagnetic pulse on (*a*) departure probability and (*b*) consistency in flight direction in juvenile and adult free-flying European robins (*Erithacus rubecula*) separated between 2020 and 2021 autumn migration (graph with further (reproducible) migratory traits, split between years can be found in electronic supplementary material, figure S3). (*a*) Departure probability represents the proportion of birds staying for the first night after treatment and release (upper bar, black numbers) or departing on the first night (lower bar, white numbers). (b) The consistency of the initial direction until passage at the shoreline of the German Bight (50–100 km, figure 1*b*).

Stopover duration of sham control juveniles was zero nights in median (1st–3rd quartile: 0–1 nights; range 0–15 nights, *n* = 56) and not different from the treatment group (1st–3rd quartile: 0–1 nights; range 0–23 nights, *n* = 53; Wilcoxon rank sum test: W = 1361, *p* = 0.382; figure 3*b*). Sham control adults also stayed zero nights in median (1st–3rd quartile: 0–8 nights; range 0–20 nights, *n* = 53), not different from pulse-treated adults (median: 1 night; 1st–3rd quartile: 0–9 nights; range 0–25 nights, *n* = 57; Wilcoxon rank sum test: W = 1299, *p* = 0.179; figure 3*b*; table 1). Pooling the age classes also reveals no effect of the pulse treatment on stopover duration (Wilcoxon rank sum test: *W* = 5275, *p* = 0.089, *n* = 219; table 1).

Nocturnal departure timing of sham control juveniles was at 22% of the night length in median (1st–3rd quartile: 16–33%; range: 8–79%; *n* = 53; figure 3*c*), not different (Wilcoxon rank sum test: *W* = 1063, *p* = 0.207; table 1) to pulsed juveniles departing after 28% of the night length in median (1st–3rd quartile: 18–41%; range: 8–71%; *n* = 47). Sham control adults departed in median at 22% of the night (1st–3rd quartile: 14–31%; range: 8–66%; *n* = 46; figure 3*c*), not different (exact Wilcoxon rank sum test: *W* = 907, *p* = 0.181; table 1) to pulse treated adults, who departed at 28% of the night length in median (1st–3rd quartile: 18–40%; range: 6–69%; *n* = 47). When pooling the age classes, we also found no difference between the treatment groups (Wilcoxon rank sum test: *W* = 3926, *p* = 0.061, *n* = 193; table 1).

Departure direction of sham control juveniles was oriented towards 122° (Rayleigh test: *r* = 0.432, *p* < 0.001, *n* = 50), but did not differ (Mardia–Watson–Wheeler test: *W* = 2.227, d.f. = 2, *p* = 0.328; table 1) from the pulsed juveniles departing to 95° (Rayleigh test: *r* = 0.348, *p* = 0.003, *n* = 46, figure 3*d*). Sham control adults were oriented towards 136° (Rayleigh test: *r* = 0.544, *p* < 0.001, *n* = 46), but did not differ (Mardia–Watson–Wheeler test: *W* = 1.365, d.f. = 2, *p* = 0.505) from the pulse treated group departing to 123° (Rayleigh test: *r* = 0.391, *p* < 0.001, *n* = 45; figure 3*d*). When pooling the age classes, we found no difference between the treatment groups either (Mardia–Watson–Wheeler test: *W* = 3.148, d.f. = 2, *p* = 0.207, *n* = 187; table 1).

The consistency of flight direction showed a mean deviation of −13° (anticlockwise) from the initial departure direction for sham control juveniles (Rayleigh test: *r* = 0.631, *p* < 0.001, *n* = 35) and −14° for pulse treated juveniles (Rayleigh test: *r* = 0.806, *p* < 0.001, *n* = 37; figure 3*d*). The spread was significantly larger for sham control juveniles (Mardia–Watson–Wheeler test: *W* = 6.023, d.f. = 2, *p* = 0.049; table 1). Sham control adults changed their flight direction by −6° (Rayleigh test: *r* = 0.870, *p* < 0.001, *n* = 35), but did not differ (Mardia–Watson–Wheeler test: *W* = 4.153, d.f. = 2, *p* = 0.126, table 1) from the treatment group changing their flight direction by −22° (Rayleigh test: *r* = 0.869, *p* < 0.001, *n* = 29, figure 3*d*). We could not find an effect when testing independent of age class (Mardia–Watson–Wheeler test: *W* = 3.184, d.f. = 2, *p* = 0.204, *n* = 136; table 1).

## 4. Discussion

In our sham-controlled study, we observed no biologically relevant effects of the magnetic treatment pulse on migratory behaviour of robins during autumn migration. This is similar to our previous work at the same study site with the same species during spring migration [31] and with northern wheatears (*Oenanthe oenanthe*) during autumn migration [30]. This is in contrast to earlier free-flight studies that also used robins in the same migration period, but in southern Germany (Lake Constance) [29], and to other comparable studies in caged experiments in central Germany (Frankfurt) [16], in Australia [7–13,15] and North America [14]. Controlling for the induced electric field (i.e. same rate of change in *B*-field in both sham and control) enables us to discuss the absence of observable effects solely in terms of the magnetic component of the pulse.

### 4.1. Multiple explanations for missing effects

The fact that we did not observe a magnetic pulse effect can have multiple explanations:

(i) The magnetic pulse did not perturb magnetic-particle magnetosensory cells strongly enough. We deem this explanation unlikely, as 52 mT pulse intensity is well above the switching field of 24 mT reported for a putative magnetoreceptor structure in trout [24].
(ii) The birds sensed the manipulation and ignored the corrupted information of the magnetic-particle-based map sense by following their innate migratory direction, using (for instance) their star or magnetic compass [2] or olfactory and landmark cues [1].
(iii) The birds obtained geomagnetic map information upon arrival at the study site prior to the pulse treatment and retained this information for their migratory decisions. However, the explanations (i)–(iii) fall short when trying to explain why other studies with free-flying birds found pulse effects [28,29].
(iv) Because the birds did not depart in the generally expected migratory direction towards SW in this region [37,38], but instead to ESE, it might be possible that some local geographical cues at the stopover site Helgoland (e.g. the coast, artificial light at night) overrode navigational decisions based on the Earth's magnetic field. Notably, it has been shown that the general migration direction along the entire migration route (broadly SW for robins along the western European flyway) can be quite different from the realised departure directions from single stopover sites. In a stopover ecology study on the natural departure behaviour of robins on Helgoland in autumn, unmanipulated birds departed towards SE (138°) on average but the departure direction varied substantially between 53° and 335° (Rayleigh test: *r* = 0.48) [45], very similar to the robins in this study. Likewise, in our former magnetic pulse study also with robins but in spring, the control birds, which were not exposed to any pulse [31], departed in the same direction as unmanipulated robins from another study on Helgoland during spring [58]. We are, therefore, convinced that our robins behaved normally and that their departure directions were not affected by the electrical component of the pulse, which both groups in this study received.
(v) The pulse magnetically ‘translocated’ the birds to a place, relative to their goal, where they did not have to compensate for this manipulation. However, we deem this explanation unlikely, as we already tested robins in spring [31] and northern wheatears in autumn [30]. Accordingly, species' migratory goals differ substantially in distance and direction, but we still found no effect of the magnetic pulse.
(vi) Finally, birds may not possess a magnetic-particle-based sensor, and sense geomagnetic map factors with the radical-pair mechanism [21,31], although this would not explain why an intact trigeminal nerve is needed to correct for a physical displacement [6].

### 4.2. Question of reproducibility

The experiments from 2020, when taken alone, prompt the conclusion that the magnetic pulse clearly had a negative effect on the migratory motivation of adult birds, represented by a decreased departure probability and increased stopover duration in the magnetic pulse treated group (table 1, figure 4). Thus, the treatment pulse may well have affected a magnetic particle-based receptor, and it is up to speculation whether the delay in departure was caused by recalibration of affected receptors or by ignoring them altogether and re-weighing other available cues in the multisensory integration process. With this effect being restricted to adults (figure 4*a*, table 1), it supports the notion of a magnetic-particle-based map-sense in experienced migrants and might have been publishable on its own. However, this effect was not reproducible in 2021 (table 1, figure 4*a*). Furthermore, after pooling the data of adult birds from both years, we found no effect (table 1, figure 4*a*).

An additional effect which occurred only in 1 year can be found in the consistency of flight direction, where sham control juveniles in 2021 were more likely to change directions than the pulse treated juveniles (figure 4*b*, table 1). This observation cannot be explained in the conceptual framework of magnetic-particle-based map-sensing and was not present in 2020 (figure 4*b*, table 1). Most likely, it represents a normal biological variation, and we have no way of knowing whether the motivational effect, discussed above, has the same origin.

Regarding instrumentation, the only difference between the 2 years was the shape of the treatment pulse with a sharper cut-off in 2020, resulting in a more pronounced induced electric field spike at the trailing edge (figure 2*c,e*). However, since the induced electric field spikes were controlled for in the sham pulse, we deem the pulse shape very unlikely to have caused the negative effects of the treatment pulse on motivation observed in 2020.

These year-specific observations, despite there otherwise being no obvious biologically relevant difference in intrinsic and extrinsic factors between the years (electronic supplementary material, table S2) and despite the large sample size, demonstrate that replication is crucial for obtaining robust results. We suggest that the high natural variation in animal behaviour makes these experiments prone to yield false positives. As a pre-Saharan migrant, our study species is known in particular for its higher within-species variation in migratory behaviour, like stopover duration or departure timing, in comparison to trans-Saharan migrants, like the northern wheatear [45]. Therefore, it is important to consider the migration ecology of the species and its associated variation in behaviour when designing future experiments and interpreting corresponding results.

### 4.3. Potential side-effects of an electromagnetic pulse

An electromagnetic pulse could potentially evoke side-effects non-specific to magnetoreception, either through the induced magnetic or the electrical field. For example, some genes that are related to repair of photoreceptive structures were upregulated in the rainbow trout when exposed to a magnetic pulse (85 mT, 5 ms) [59]. Another example of pulse effects comes from cell cultures, where pulsed magnetic fields (multiple low intensity oscillating magnetic fields) are intentionally used to affect regulatory pathways, e.g. [60]. A further side-effect might occur through iron-rich structures in the beak of birds, not related to magnetoreception [26]. An electromagnetic pulse will alter these structures and may evoke a response in the bird. Any of these potential side-effects would be independent of age and unrelated to magnetoreception. Whether our study may tend towards this (see last column of table 1 with 0.1 > *p* > 0.05) remains speculative.

### 4.4. Perspectives and conclusion

With our series of pulse experiments ([30,31] and this study), we originally intended to answer in-depth questions about the magnetic-particle-based map sense in songbirds, but unlike similar previous studies, we did not find an effect [28,29]. To resolve these contradictory findings, the actual magnetic-particle-based receptor should be found so that its structure and corresponding neuronal magnetoreception pathways can be understood. Without knowing any specifics, we cannot reliably predict the receptor's reaction to a pulse and draw conclusions from a bird's behaviour after receiving a pulse treatment. This becomes especially apparent as the pulse does not seem to result in predictable changes in behaviour, with disorientation, unimodal and bimodal reorientation all seen in previous cage and field experiments, often depending on the orientation of the pulse [15,16,28,29]. As finding the sensor structure is a tedious and probably lengthy task, e.g. [26], we propose a series of potential future experiments to shed more light on this matter.

(I) Methodologically, we propose using a pulser device that enables controlling for potential effects of the electric field component, to conduct experiments with a reasonable high sample size, ideally over more than 1 year, and to collect and analyse data free from any observer bias.
(II) We need independent, fully blinded replications of orientation-cage-based pulse experiments under meticulously controlled conditions in the laboratory, to validate the basic hypothesis of magnetic-particle-based magnetoreception [7–16].
(III) Thus far, pulse experiments with songbirds were predominantly conducted at sites where we do not know if birds relied on magnetic map information for their migratory decisions. To carefully approach a scenario in which birds probably access navigational map information, we propose translocating birds, either physically or virtually [18,20,21], to a novel location. Provided they show corrective orientation tendencies toward the migratory destination in orientation cages, i.e. correcting for the translocation, their orientations should be tested again, now after a pulse treatment. If corrective orientation tendencies are maintained in the sham group but aborted in the treatment group, the case for a magnetic-particle sensor can be made.
(IV) If successful, the post-translocation pulsing experiment should be expanded from caged to free-flying birds to investigate the importance of this sensor information in the wild. This translocation must ideally be performed at a site without ‘any’ geographical constraints, such as coastlines or open ocean, lakes, rivers, mountain ridges, artificial light (e.g., cities, streets, towers) or patchily distributed habitats, which may override other navigational decisions.
(V) If virtual translocation experiments (III + IV) do not reveal any evidence for the magnetic-particle-based sensor, we may have to consider an alternative sensor for magnetic map navigation, specifically the radical-pair-based mechanism of magnetoreception sensor [21,31].

## Data Availability

The datasets and codes supporting this article have been uploaded as part of the electronic supplementary material [61].
